# Humor and Fear—Two Sides of the Same Coin?: Experimental Evidence on Humor Appeals in Health Communication Related to Childhood Vaccination

**DOI:** 10.3389/fpubh.2021.649507

**Published:** 2021-04-27

**Authors:** Florian Fischer, Franziska Carow, Stefanie Gillitzer

**Affiliations:** ^1^Institute of Public Health, Charité—Universitätsmedizin Berlin, Berlin, Germany; ^2^Institute of Gerontological Health Services and Nursing Research, Ravensburg-Weingarten University of Applied Sciences, Weingarten, Germany; ^3^School of Public Health, Bielefeld University, Bielefeld, Germany

**Keywords:** humor, health, communication, science, herd immunity, vaccine, emotion

## Abstract

Until now, health communication has largely failed to debunk fears and caveats related to vaccination. This study aims to investigate the effects of different text types used in health communication in an experimental study design. A neutrally formulated text was compared to a humorous text using the formula of a fairytale. Overall, the study indicates no additional value in using the humorous format as an innovative and target-group-oriented approach to inform readers about scientific evidence related to vaccination. Although the effects of the two text types do not differ, the credibility of the neutrally formulated text was much more likely to be judged as high. This indicates that the perception of credibility is not the only criterion in health communication leading to knowledge gains and changes in health-related attitudes and behaviors.

## Introduction

The main goals of health communication are empowerment for health-related decision-making and fostering health literacy (1, 2). However, health-related issues are frequently highly controversial, which leads to specific challenges for health communication. One such example is vaccination. Despite widespread evidence for the safety and effectiveness of vaccinations, low acceptance rates still interfere with the eradication of vaccine-preventable diseases (3, 4). Vaccination opponents propagate false information and “alternative facts,” leading to irrational fears among the public, which run counter to the current state of scientific evidence (5, 6). The communication of risks and uncertainties related to vaccinations—as a further trigger for fears—have also been proven to be a particular challenge during the COVID-19 pandemic (7). However, previous research has also emphasized the need for adequate vaccine health communication—and the challenges associated with it (8, 9).

Vaccination is a highly emotional and currently much-discussed topic. Therefore, it seems to be an appropriate time to describe the relevance of emotions in health communication. Until now, health communication has failed to debunk the fears and caveats related to vaccination (10). Vaccination targets are to reach a specific vaccination coverage range among the population in order to gain the benefits of herd immunity (11). Herd immunity, also referred to as community immunity (12), is a form of indirect protection from infectious disease that occurs when a large percentage of a population has become immune to an infection, thereby providing a measure of protection for individuals who are not immune. Most vaccines protect both vaccinated individuals and the society in which they live. Therefore, vaccination is a prosocial act, and can even be seen as a social contract (13). It has been shown that communicating the concept of herd immunity leads to greater willingness to be vaccinated, particularly in cultures lacking this prosocial cultural background (9).

In a population in which a large number of individuals are immune, chains of infection are likely to be disrupted, which stops or slows the spread of disease (14). Despite the full insurance coverage of childhood vaccinations in Germany, there is still a degree of vaccination hesitancy, which displays regional variations and differences according to socio-economic status (15, 16). Knowledge about the vaccine, social influences, and trust in health care are factors affecting vaccine uptake in young children (10). Explanations for differences in vaccination uptake can be found in the federal structure and, therefore, in the different historical developments of vaccinations and the lack of a common strategy in the organization of public health services (17). However, primarily there are anti-vaccination beliefs leading to an overestimation of complications due to insufficient, confusing, or even incorrect information.

Another explanation for not vaccinating one's children can be an underestimation of a disease's consequences. Furthermore, people show a tendency to appraise the risk of a hazard based on their own feelings. For example, people generally evaluate human hazards more negatively than natural hazards (“affect-heuristic”) (18). In regard to vaccination, people might be afraid that a humanity-produced vaccine might have more adverse effects than the disease itself. In addition to this, the negative evaluation of an uncommunicated risk is less strong than the negative appraisal of a communicated risk (19). In terms of vaccination, this means: talking about even very rare adverse effects of a vaccination is assessed more negatively than disease symptoms that people do not know about. Even if the objectively judged risk of the effects of the disease is much higher, the appraisal of the potential side-effects of the vaccination is weighted more heavily. This phenomenon is known as “negativity bias” (17). Previous research has highlighted that providing statistical information is not as influential as narrative information (8). High (vs. low) emotional narratives have a greater impact on the perceived risks related to vaccination (20).

In Germany, there has been a strong statement from the national ethics committee against mandatory measles vaccinations for reasons of valuing freedom of choice more highly than social welfare (21). However, as of March 2020, parents must prove that their child has been vaccinated against measles before they can send them to daycare or school. This is an exception to the otherwise voluntary vaccinations. Previous research in other areas has shown that decreasing people's freedom of choice may result in reactance (22, 23). This emphasizes the need for effective communication strategies about the advantages of vaccination, such as herd immunity, in order to increase vaccination intentions (12). Experimental evidence shows the positive effects of communication about herd immunity on vaccination intentions and its impact on buffering the reactance effects of selective mandatory vaccinations (24). A study comparing a satirical message to a more serious message on measles vaccination revealed that the use of humor reduced reactance and led to greater understanding of the severity of measles, which reduced vaccine hesitancy (25). Furthermore, evidence suggests that the effectiveness of communication about fear-related topics can be increased by adding an element of humor (26).

Considering the emotions that are evoked by many health-related topics, one needs to find adequate strategies in health communication to inform people of the evidence and debunk those fears, such as by the use of humor (27). Within the past few years, entertainment education, which consists of systematic storytelling, has been discovered as a new way of communicating health-related topics, such as vaccination (28). In these narratives, scientific knowledge can be combined with emotions, so that the reader is able to identify with or feel empathy for the story's characters. Research shows that the content and structure of stories have a positive influence on the understanding and retention of information. If a topic is not interesting enough, a story is able to evoke interest and the ability to remember its content in the long term. The chronological structure of a story can help people to assimilate even abstract subjects (29, 30).

This study aims to analyze how different text types used in health communication about vaccination—one formulated in a neutrally scientific way and the other as a humorous text applying the format of a fairytale—are appraised by the recipients. Furthermore, the impacts of both text types on the knowledge and attitudes of parents toward childhood vaccinations are investigated.

## Methods

An experimental study was conducted among parents having at least one child of kindergarten age (up to 6 years old). Recruitment took place in kindergartens in Bielefeld (a city with about 330,000 inhabitants in Germany) and using social media (Facebook). The sample was primarily collected in kindergartens in Bielefeld, because it can be assumed that within the different kindergartens—according to the residential area and the funding body—the heterogeneity in terms of sociodemographic characteristics of all parents is represented. Therefore, we expected to reach an almost representative sample representing the majority of parents of small children in Bielefeld. Paper-and-pencil questionnaires were used in the kindergarten setting, while the same questionnaire was used in an online format for the participants recruited via social media.

Study participants filled out a questionnaire that was specifically developed for this study (Supplementary Appendix 1), received an intervention directly afterwards in the form of a written text informing them about vaccination (in particular, the concept of herd immunity), and were asked some questions related to this text. A neutrally formulated text (Supplementary Appendix 2a) published by the Federal Centre for Health Education (BZgA), which is a specialist authority within the portfolio of the Federal Ministry of Health in Germany aiming to promote health education (e.g., in terms of guidelines, training, and coordination) at the national level, was compared to a text written by Dr. Eckart von Hirschhausen, a famous cabaret artist and comedian, which used humorous elements in the form of a fairytale (Supplementary Appendix 2b). The texts are both written in the German language, include the same arguments, and are comparable in length (439 vs. 426 words). The authors of the texts are not mentioned. One of the two texts was randomly allocated to each study participant. For the paper-and-pencil questionnaire, the randomization took place at the level of kindergartens (with the parents of children within one kindergarten all receiving the same intervention), and for the online questionnaire at the individual level (with random allocation). No compensation was provided for participation.

Standardized tools were used to assess socio-demographic variables (age, sex, marital status, family structure, education, and migration background), vaccination behavior and the parents' reasons for vaccination, risk perception, trust in vaccination recommendations by the Standing Committee on Vaccination (STIKO), and knowledge related to childhood vaccinations (pre- and post-test). In addition to measuring the intervention's effects on vaccination attitudes, we assessed questions related to the comprehensibility, credibility, and learning effect of the texts.

Descriptive and bivariate analyses were conducted. Differences between the two texts were assessed using the *t*-test for independent samples. The significance level was set at *p* < 0.05. All statistical analyses were carried out using the statistics software IBM SPSS Statistics 25. The study received a positive ethics vote from the ethical committee of Bielefeld University.

## Results

### Sample Characteristics

The sample consisted of 120 participants who completed the questionnaire either in the paper-and-pencil form (*n* = 40; 33.3%) or the online version (*n* = 80; 66.7%). The neutrally formulated text was read by 66 people (55.0%), while 54 people (45.0%) received the humorous text as their intervention. There are no significant differences in sample characteristics between the two different methods of data collection, or between the two interventions.

The total sample was predominantly female (*n* = 102; 85.0%). More than half of study participants were aged 30 to 39 years (*n* = 67; 55.8%). Seventeen (14.2%) had immigrated to Germany, although they had been living in Germany for at least seven years. Among the interviewees, about 81% (*n* = 97) were married, 13% (*n* = 16) were in a partnership, and 6% (*n* = 7) were single parents. The sample shows an overall high level of education, because more than half (*n* = 63; 52.5%) of the participants had a University degree, and 47.5% (*n* = 57) had a secondary school exam qualifying them for University admission (Table 1).

**Table 1:** Sample characteristics (*n* = 120).

		**Total**		**Neutral**		**Humor**		
		***n***	**%**	***n***	**%**	***n***	**%**	***P*-value**
Sex	Male	18	15.0	9	13.6	9	16.7	0.798
	Female	102	85.0	57	86.4	45	83.3	
Age	Up to 29 years	20	16.7	13	19.7	7	13.0	0.602
	30 to 39 years	67	55.8	36	54.5	31	57.4	
	40 years and more	33	27.5	17	25.8	16	29.6	
Marital status	Married	97	80.8	54	81.8	43	79.6	0.908
	Partnership	16	13.3	8	12.1	8	14.8	
	Single parent	7	5.8	4	6.1	3	5.6	
Education	Secondary education	57	47.5	34	51.5	23	42.6	0.604
	University degree	63	52.5	32	48.5	31	57.4	

### Information, Knowledge, Attitudes, and Behaviors Related to Vaccination

Overall, we found a high positive attitude toward vaccination. This is true for both kinds of intervention, with 83.9% (*n* = 55) in the group reading the neutral text and 80.8% (*n* = 43) of the participants who read the humorous text indicating a positive attitude toward vaccination. In addition, 75.6% (*n* = 90) felt well or very well-informed about vaccination in general, while 24.4% (*n* = 29) felt less well or rather badly informed.

The appraisal of two statements was used to assess knowledge related to the concept of herd immunity. Only half of the study participants agreed with the following statement: “If almost all people are vaccinated against a disease, it can succeed in eradicating this disease.” The assertion that one's own child is unlikely to become ill when most other children are vaccinated was fully agreed with by 19.2% (*n* = 23) of parents. Both values are comparatively low, indicating missing knowledge related to herd immunity.

The study participants were asked to mention all the channels they had used for gaining information about vaccination. Most of the respondents claimed that they receive information related to vaccination from their physician (88.3%; *n* = 106). The second most commonly used information source was websites of institutions related to health, such as the BZgA, mentioned by about 37.5% (*n* = 45). About a quarter or fewer also mentioned the following information sources: friends (23.3%; *n* = 28), the internet in general, social media, or blogs (22.5%; *n* = 27); and newspapers or journals (22.5%; *n* = 27). Information was also retrieved from books (20.0%; *n* = 24), health insurance (14.2%; *n* = 17), and television (6.7%; *n* = 8).

The majority of respondents indicated a positive attitude toward vaccination (82.2%; *n* = 83). According to the self-reported information provided by the parents, 48.3% (*n* = 58) of children had an incomplete vaccination status; that is, fewer than seven out of nine vaccinations. Overall, 15.0% (*n* = 18) of the children were fully vaccinated and 10.8% (*n* = 13) were not vaccinated at all. Overall, there were no significant differences between the two groups in terms of sociodemographic characteristics, information, knowledge, attitudes, or behaviors related to vaccination.

### Appraisal of the Texts

The study participants were asked to appraise the text they had been given. The *t*-test showed no significant differences in the appraisal of the two texts in terms of comprehension, learning effect, or opinion-forming. The appraisal of the credibility differed slightly, but not significantly, between the texts. However, a higher proportion of participants who read the neutrally formulated text by BZgA claimed a desire to read more texts of this kind (66.2%; *n* = 43), compared to those who read the humorous text (46.2%; *n* = 24).

Comprehension was rated very high for both texts, with 96.9% (*n* = 62) of the readers of the neutral text stating that they understood it, while 96.2% (*n* = 50) of the readers of the humorous text said the same. There was only a slight difference in the appraisal of credibility: 93.8% (*n* = 61) considered the neutral text to be credible and 84.3% (*n* = 43) said the humorous text was credible (Table 2).

**Table 2:** Appraisal of the texts (*n* = 108).

	**Neutral**		**Humor**		**Chi square Pearson**	***T***	***P*-value**
	**(*****n*** **=** **65)**		**(*****n*** **=** **53)**				
	***n***	**%**	***n***	**%**			
Did you understand all aspects of the text?	62	96.9	50	96.2	χ^2^(1) = 0.045 (*p* = 0.832)	1.084	0.281
Do you think the information in the text is credible?	61	93.8	43	84.3	χ^2^(1) = 2.800 (*p* = 0.094)	1.408	0.162
Would you like to read more such texts about vaccination or other health issues?	43	66.2	24	46.2	χ^2^(1) = 4.722 (*p* = 0.030)^*^	2.413	0.017^*^
Have you learned anything new about vaccination from the text?	21	32.3	9	17.6	χ^2^(1) = 3.204 (*p* = 0.073)	0.838	0.404
Did the text support you in forming an opinion about vaccination?	21	32.3	12	23.5	χ^2^(1) = 1.195 (*p* = 0.274)	0.499	0.619

* *p < 0.05*.

Small to medium correlations were observed between the different categories for appraising the texts. The credibility and the desire to read more texts of this kind correlated moderately (*r* = 0.333; *p* < 0.001). Furthermore, there was a correlation between the question of whether something new had been learned from the text and the statement that the text could influence one's opinion on the subject of vaccination (*r* = 0.411, *p* < 0.001). Understanding the context of the text was significantly correlated with the judgement of its credibility (*r* = 0.246; *p* = 0.008).

Study participants were asked whether they felt more or less willing to vaccinate their children after reading the text. Three quarters of the study participants in both groups stated no change in their intention to vaccinate their children. After reading the neutral text, 23.1% (*n* = 15) expressed a greater willingness to vaccinate. This compares to 21.6% (*n* = 11) of the respondents in the group who read the humorous text [χ^2^(2) = 0.848 (*p* = 0.654)], indicating no difference in the effects of the two texts on vaccination attitudes.

## Discussion

The study results indicate no additional value in using the format of a humorous text in the form of a fairytale as an innovative and target-group-oriented approach to informing readers about scientific evidence related to vaccination. However, questions emerge as to why the effects between the two text types do not differ, despite the fact that the credibility was judged to be much higher in the neutrally formulated text. This indicates that the perception of credibility is not the only factor in health communication leading to knowledge gains.

Investigating the effects of health communication related to vaccines—as conducted within this study—is very important for several reasons: the latest research has shown that trust in science and also trust in doctors, scientific institutions, and governments has decreased (31, 32). Different factors may lead to less trust, such as the rapid development of the internet and social media, the complexity and pace of information available (33), and a rising motivation to reject science among certain groups in society (34). Decreased trust in science, institutions, and authorities may lead to uncertainty in individuals and, thus, to different (i.e., not health-promoting) behavior. For this reason, as well as the implications of the spread of fake news and social media, a new or different way of communicating scientific evidence about health-related issues becomes relevant (33). A recent example of increasing anti-vaccination behavior in Italy, which led to increasing numbers of measles outbreaks, demonstrates one of the main challenges: a movie about vaccination against measles spread fake news. In this movie, it is claimed that autism is supposed to be an adverse effect of the vaccination against measles, mumps, and rubella. This movie, in addition to other reports carried by different media, led to insecurity about the risk of vaccination against measles in society, and to different vaccination behavior (35). A previous study has shown that narratives have a stronger impact than statistical risk information on perceived vaccination risks. Furthermore, the same study emphasized that the number of narratives reporting adverse effects related to vaccination is inversely related to vaccination intentions (20). This indicates the relevance of information that goes beyond neutrally formulated texts. However, the texts themselves need to be rated as credible. In terms of the credibility, effects, and impact of science communication in the field of public health, the investigation of new and different forms of health communication is not only worthwhile but necessary. Using innovative approaches, such as including humor in communication strategies, may enable communicators to reach a wider audience (36).

For example, the use of humor in health communication about fear-related topics might be successful, because the tailored communication strategies using humor can be used to work with—rather than against—these underlying fears (34). In contrast to the results of this study, it has been shown that humorous elements incorporated into health communication might be beneficial for other topics which are also related to actions, such as organ donation (37, 38). Among psychological coping strategies—beyond denial, defensive avoidance, and reactance—humor has a promising advantage: previous research has validated that the use of humor supports people in coping with stress, anxiety, and fear (39), which are all potential responses to fear appeals (40). As already suggested by Steindl et al. (41), it would be interesting to take a closer look at the association between reactance and negative emotions (e.g., fear) and positive emotions (e.g., humor).

Although some studies have already explored the advantages of narrative texts, they are obviously not suitable for conveying every subject. Narratives do not usually contain explicitly formulated arguments for or against a fact and are therefore less instructive (42). However, it should be borne in mind that people who read a text on vaccination for information purposes want to see arguments and facts to facilitate their decision for or against vaccination. This is particularly important in relation to the autonomy of parents. Since parents want to decide autonomously about the welfare of their children, patronizing information can lead to a rejection of the issue. There is a risk that a narrative story will distract from the relevant information if the plot and the information to be conveyed are not sufficiently closely linked (42).

Based on our results focusing on vaccination among children, we conclude that the use of storytelling, using emotional phrases or content, in the field of health communication needs to be tailored toward the recipients. In our case, it seems as though the description of true events or texts written in such a way that it is possible to identify with the narrative leads to higher levels of confidence. The concept of entertainment education, as it is already used in other countries, is not primarily about providing a range of information, but rather about attracting attention to a topic. Recipients should be made aware of topics through the entertainment they have already enjoyed (43). The aim behind this concept is thus an educational one. Therefore, the primary task of the humorous text within our study is to arouse interest in the topic of vaccination and, thereby, to increase the health literacy of recipients. It would be ideal if readers were then to call the topic of vaccination into their consciousness and consequently inform themselves more deeply about it.

## Limitations

This study faces several methodological limitations. First of all, the results are based on a small sample size. The sample lacked a denominator, so we were not able to calculate a response rate. It needs to be emphasized that all the information gathered is based on the self-reported judgement of the study participants themselves. The results should be interpreted with caution due to ceiling effects: the understandability of both texts was very high. Therefore, one cannot investigate the impact of different text styles on comprehensibility, which might affect knowledge and/or behavior. Future research in this area might assess the functional health literacy related to this topic. The ceiling effects might, therefore, have led to an underestimation of effects, particularly because—according to the self-reported appraisal—the humorous text was not judged to be equally as credible as the neutral text.

Furthermore, the findings are not generalizable, because a very specific format of humorous text, in the form of a fairytale, was used. We did not include manipulation checks to see whether participants perceived these two messages in the way we expected. Future research in this area should use fact-recall items or other forms of factual knowledge indicators. In addition, we focused on measuring affective reactions to the stimulus material, such as how humorous the study participants judged the material to be, or how much they enjoyed reading it. One needs to acknowledge that the texts were not tailored to the target group; for example, they did not explicitly focus on young children.

No subgroup analyses were conducted, although it might be interesting to focus on differences in the intervention's impact between those in favor of childhood vaccinations and those who are hesitant. However, the sample was too small for any analyses of this kind.

## Conclusions

Previous research has provided evidence that the use of humor in health communication may lead to changes in attitudes and behaviors. This is of particular relevance for topics associated with irrationality and fear, such as vaccination. Although this study did not reveal any benefit of the humorous format of health communication compared to the traditional format, it should be considered that the appraisal and impact of both text types were almost equal. This result is important, because the humorous presentation might attract more interest and attention from the public than a neutrally or scientifically formulated text.

## Data Availability Statement

The raw data supporting the conclusions of this article will be made available by the authors, without undue reservation.

## Ethics Statement

The studies involving human participants were reviewed and approved by Ethics committee of Bielefeld University. The patients/participants provided their written informed consent to participate in this study.

## Author Contributions

FF and SG conceptualized the study. SG collected and analyzed the data. FF supervised this process and drafted the manuscript. FC and SG revised the manuscript critically for important intellectual content. All authors approved the final manuscript.

## Conflict of Interest

The authors declare that the research was conducted in the absence of any commercial or financial relationships that could be construed as a potential conflict of interest.
