# Supplementary material for: Humor and Fear—Two Sides of the Same Coin?: Experimental Evidence on Humor Appeals in Health Communication Related to Childhood Vaccination
Source: Front Public Health. 2021 Apr 27;9:649507. doi: 10.3389/fpubh.2021.649507 (PMC8112543; doi:10.3389/fpubh.2021.649507)
Supplement: Supplementary file 1 [file Data_Sheet_1.PDF]

Supplementary Appendix 1: Questionnaire

All items relevant for our study are shown below in a translated version. The original questionnaire was in German language.

Sex/gender

**O**	Male
**O**	Female

Marital status

**O**	Married
**O**	Living in a partnership
**O**	Single parent

How old are you and your partner?

Please enter the age of your partner only if you have previously checked "Married" or "Living in a partnership".

**Age: ________________**
**Age of your partner: ________________**

How many children live in your family? Please indicate how old the children are.

Please write behind the number 1. the age of your first-born child, behind the number 2. the age of your second child and so on.

**1.**	**7.**
**2.**	**8.**
**3.**	**9.**
**4.**	**10.**
**5.**	**11.**
**6.**	**12.**

Were you or your partner born in a country other than Germany?

Multiple answers possible. If you indicated "Single parent" above, please enter only the information about yourself.

**O**	Yes, myself.
**O**	Yes, my partner.
**O**	No.

How long are you already living in Germany?

Please enter the years you have lived in Germany. If you have lived in Germany for 3 years and 7 months, write "4". If you were born in Germany, please skip this question.

**Years in Germany: ________________**

What is your highest educational qualification?

**O**	No qualification
**O**	Graduation from a special school
**O**	Primary school
**O**	Secondary school
**O**	University degree
**O**	Other: ____

How well do you feel informed about vaccination?

Very good	Good	Less good	Rather bad	Not at all
**O**	**O**	**O**	**O**	**O**

Where do you mainly get information about vaccinations?

Multiple answers possible.

**O**	Doctor/ Physician
**O**	Nurse
**O**	Newspapers and magazines
**O**	Books
**O**	Kindergarten
**O**	Television
**O**	Friends and acquaintances
**O**	Internet or social media, blogs
**O**	Specialized sites on the internet (e.g. BZgA, RKI...)
**O**	Health insurance
**O**	Others, namely:

To what extent do you agree with the following statements?

Please provide an assessment for ***each*** *statement.*

	Agree	Tend to agree	Tend to disagree	Disagree
**“If almost all people are vaccinated against a disease, it can succeed in eradicating this disease.”**	O	O	O	O
**“If most other children are vaccinated, it is unlikely that my child will get one of those diseases.”**	O	O	O	O
**“Some vaccines are more dangerous than the disease they are supposed to protect against.”**	O	O	O	O
**"There are alternative methods that are more effective than vaccines.”**	O	O	O	O
**“If a child is fed a healthy diet, he or she doesn't need vaccinations.”**	O	O	O	O

--- Intervention: Either neutrally formulated or humorous text ---

Now please answer the following questions in relation to the text. Then you have done it.

	Yes	Rather yes	Rather no	No
**Did you understand all aspects of the text?**	O	O	O	O
**Do you think the information in the text is credible?**	O	O	O	O
**Would you like to read more such texts about vaccination or other health issues?**	O	O	O	O
**Have you learned anything new about vaccination from the text?**	O	O	O	O
**Did the text support you in forming an opinion about vaccination?**	O	O	O	O

Supplementary Appendix 2: Interventions
2a) Neutrally formulated text

Original version in German language

Herdenimmunität: Schutz für den Einzelnen und die Gemeinschaft
Durch Impfungen schützt man sich in erster Linie natürlich selbst vor den ansteckenden Krankheiten. Doch es gibt auch Menschen, die sich nicht impfen lassen können. Für manche Impfungen sind Babys beispielsweise noch zu jung, andere Menschen können aufgrund einer chronischen Erkrankung die eine oder andere Impfung nicht bekommen. Sie sind darauf angewiesen, dass die Menschen in ihrem Umfeld geimpft sind und ihnen Schutz vor der Ausbreitung und Ansteckung mit der Krankheit bieten. Man spricht dann von Herdenimmunität. Der eigene Impfschutz trägt also gleichzeitig zum Schutz der Gemeinschaft bei. Lassen sich ausreichend viele Menschen impfen, so kann für einige Krankheiten sogar verhindert werden, dass sie weiterhin auftreten.
Das Beispiel: Kinderlähmung
Viele Erkrankungen, die in Deutschland nur noch selten vorkommen, sind in anderen Teilen der Welt noch immer verbreitet. Ein Beispiel ist die Kinderlähmung (Polio). Die Krankheit gilt in ganz Europa und auf dem amerikanischen Kontinent zwar als ausgerottet. Trotzdem ist es auch heute noch wichtig, dass weiterhin gegen Kinderlähmung geimpft wird. Denn würde die Kinderlähmung, die in Teilen Asiens und Afrikas weiterhin vorkommt, zum Beispiel durch Reisende nach Deutschland gelangen und auf eine weitgehend ungeimpfte Bevölkerung treffen, wäre eine erneute Ausbreitung möglich. Um einen Ausbruch in Deutschland zu verhindern, wird mit einer hohen Impfquote in der Gesamtbevölkerung die Herdenimmunität aufrechtgehalten.
Elimination der Masern und Röteln
Die Weltgesundheitsorganisation (WHO) hat sich zum Ziel gesetzt, die Verbreitung von Masern und Röteln weltweit zu verhindern. Dies ist möglich, wenn langfristig 95 Prozent der Bevölkerung gegen diese Krankheiten geschützt sind. Durch den Impfschutz der Gemeinschaft (Herdenschutz) wären dann auch Menschen vor einer Ansteckung geschützt, die nicht geimpft werden können, - darunter beispielsweise junge Säuglinge.
In einigen Regionen der Welt sind Masern aufgrund hoher Impfquoten heute so selten, dass sie als masernfrei gelten, so zum Beispiel Nord- und Südamerika. In Deutschland ist das WHO-Ziel noch nicht ganz erreicht. So ist es in Deutschland in den vergangenen Jahren immer wieder zu regionalen Masernausbrüchen gekommen. Dabei steckten sich vermehrt auch Jugendliche und Erwachsene bis etwa Mitte vierzig an. Für Kinder und Jugendliche sind zwei Impfungen gegen Masern und Röteln empfohlen. Die Impfung erfolgt mit einem Impfstoff gegen Masern, Mumps und Röteln (MMR-Impfstoff). Während die Impfquote für die erste Masern-Impfung bei Kindern kurz vor der Einschulung zwar schon die 95 Prozent-Marke erreicht hat, fehlt die 2. Impfung allerdings noch häufiger. Es wird empfohlen, fehlende Impfungen möglichst bald nachzuholen.
Auch Erwachsene, die nach 1970 geboren sind und in der Kindheit noch keine oder nur eine Impfung gegen Masern bekommen haben, sollten sich noch einmal gegen Masern impfen lassen. Der eigene Impfschutz kann schnell durch einen Blick in den Impfpass überprüft werden.

English translation

Herd immunity: Protection for the individual and the community

Vaccinations primarily protect people from infectious diseases themselves, of course. But there are also people who cannot be vaccinated. For example, babies are too young for some vaccinations, and other people cannot get one or another vaccination because of a chronic illness. They rely on the people around them to be vaccinated and provide them with protection against spreading and contracting the disease. This is known as herd immunity. Thus, one's own vaccination protection simultaneously contributes to the protection of the community. If a sufficient number of people are vaccinated, some diseases can even be prevented from continuing to occur.

The example: Polio

Many diseases that are rare in Germany are still widespread in other parts of the world. One example is polio. The disease is considered eradicated throughout Europe and the Americas. Nevertheless, it is still important today to continue vaccinating against polio. This is because if polio, which still occurs in parts of Asia and Africa, were to reach Germany through travelers, for example, and encounter a largely unvaccinated population, it could spread again. To prevent an outbreak in Germany, herd immunity is maintained with a high vaccination rate in the overall population.

Elimination of measles and rubella

The World Health Organization (WHO) has set itself the goal of preventing the spread of measles and rubella worldwide. This is possible if 95 percent of the population is protected against these diseases in the long term. Community vaccination protection (herd protection) would then also protect people from infection who cannot be vaccinated – including young infants, for example.

In some regions of the world, measles is now so rare due to high vaccination rates that they are considered measles-free, such as North and South America. In Germany, the WHO target has not yet been fully achieved. In recent years, there have been repeated regional measles outbreaks in Germany. Adolescents and adults up to their mid-forties have also been increasingly infected. Two vaccinations against measles and rubella are recommended for children and adolescents. The vaccination is given with a vaccine against measles, mumps and rubella (MMR vaccine). While the vaccination rate for the first measles vaccination in children has already reached the 95 percent mark shortly before school enrollment, the second vaccination is still missing more frequently. It is recommended that missing vaccinations be made up as soon as possible.

Adults born after 1970 who received no vaccination or only one vaccination against measles in childhood should also be vaccinated again against measles. One's own vaccination protection can be quickly checked by looking at the vaccination certificate.

2b) Humorous text

Original version in German language

Ein Impfmärchen mit Happy End

In dem brandenburgischen Dorf Stikow wohnen 100 Leute. Von denen haben sich alle impfen lassen. So wie früher. War ja nicht alles schlecht.

Nichts ist perfekt. Auch eine wirksame Impfung erreicht nicht bei allen das gleiche Ergebnis. Angenommen, eine Impfung schlägt bei 5 Prozent nicht an, dann bleiben von den 100 Geimpften trotzdem 5 übrig, die sich noch anstecken könnten, allerdings nicht in Stikow, weil die 95, bei denen die Impfung angeschlagen hat, weder die Krankheit bekommen noch sie weitergeben können.

Es kommen 5 aus dem Nachbardorf Mumpitz zum Kaffee nach Stikow. In Mumpitz leben alle sehr gesundheitsbewusst und meinen, keine Impfung zu brauchen. Scheinbar zu Recht, denn momentan ist auch keiner krank.

Aber was passiert, wenn in diese heile Welt eine Infektionsquelle von außen einbricht? Dummerweise hat der nette Briefträger Peter Ziege, den alle nur den Ziegenpeter nennen, sich auf der Arbeit irgendwo Mumps eingefangen. Er hat heute für jeden in Stikow eine Postwurfsendung dabei und liefert gleichzeitig mit einem Auswurf hustend die Erreger ganz frisch in jeden Haushalt. Zack werden tatsächlich, obwohl doch alle geimpft sind, 5 Leute aus Stikow krank und auch die 5 ungeimpften Besucher aus Mumpitz.

Von den 10 Erkrankten sind also die Hälfte Geimpfte. Spricht das jetzt gegen eine Impfung? Nein! Natürlich nicht.

Denn die absoluten Zahlen 5 zu 5 müssen ins Verhältnis gesetzt werden zu der Grundgesamtheit, und dann liest sich die Geschichte ganz anders: Von 100 Geimpften wurden 5% krank, aber alle von den 5 Ungeimpften, also 100 Prozent. 95 Menschen von 100 wurden durch die Impfung geschützt.

Aber was passiert, wenn die Mumpitzer von ihrem infektiösen Ausflug nach Hause zurückkommen? Sie stecken auch noch die 95 anderen Daheimgebliebenen an. Und jetzt steht es zwischen den Dörfern und Überzeugungen 100 zu 5. 100 Kranke in Mumpitz. Nur 5 in Stikow.

Und wenn sie nicht gestorben sind, wissen jetzt alle in Mumpitz, dass sie von nun an gerne alle empfohlenen Impfungen wahrnehmen.

Wenn es Ausbrüche einer Infektion gibt, werden unter den Erkrankten immer auch ein paar sein, die geimpft waren. Das ist normal und spricht nicht gegen Impfungen, sondern gerade dafür. Keine Impfung ist zu 100 Prozent effektiv. Aber die Klassiker wie die Impfungen gegen Masern, Mumps und Röteln sind zu 95 Prozent und mehr wirksam.

Happy End!

Und wie kann das Happy End noch getoppt werden? Nicht nur ganz Mumpitz ist überzeugt, sondern auch der Briefträger überzeugt seine Kollegen, dass besonders für Menschen, die viel Kontakt mit anderen haben, gilt: Ich möchte andere Menschen nur mit meiner guten Laune anstecken – mit nichts sonst.

English translation
(Note: Not all humorous elements are visible in the English version due to specificities in language, e.g. the name of the village "Stikow" refers to the Standing Committee on Vaccination (STIKO) and the German term for the village "Mumpitz" is equal to "hook" and very close to the disease mumps. Furthermore, "Ziegenpeter" is the colloquial term for mumps.)

A vaccination fairytale with a happy ending

100 people live in the village of Stikow in Brandenburg. All of them have been vaccinated. Just like in the old days. Not everything was bad.

Nothing is perfect. Even an effective vaccination does not achieve the same result in everyone. Assuming that a vaccination does not work in 5 percent of the 100 vaccinated people, there are still 5 who could still be infected, but not in Stikow, because the 95 who have been vaccinated can neither get the disease nor pass it on.

There are 5 from the neighboring village of Mumpitz who come to Stikow for coffee. In Mumpitz, everyone is very health-conscious and thinks they don't need a vaccination. Apparently rightly, because at the moment no one is sick either.

But what happens when a source of infection from outside breaks into this perfect world? Stupidly enough, the nice letter carrier Peter Ziege, whom everyone just calls Ziegenpeter, has caught mumps somewhere at work. Today, he has a mailshot for everyone in Stikow, and at the same time, coughing with a sputum, he delivers the pathogens fresh to every household. Zack actually, although all are vaccinated, 5 people from Stikow become ill and also the 5 unvaccinated visitors from Mumpitz.

So half of the 10 sick people are vaccinated. Does this speak against vaccination? No! Of course not.

Because the absolute numbers 5 to 5 must be put in relation to the basic population, and then the story reads quite differently: 5% of 100 vaccinated people became ill, but all of the 5 unvaccinated people, i.e. 100 percent. 95 people out of 100 were protected by the vaccination.

But what happens when the mumpers return home from their infectious outing? They also infect the 95 others who stayed at home. And now it is 100 to 5 between the villages and convictions. 100 sick people in Mumpitz. Only 5 in Stikow.

And if they didn't die, now everyone in Mumpitz knows that from now on they will gladly take all recommended vaccinations.

When there are outbreaks of infection, among the sick there will always be a few who were vaccinated. This is normal and does not argue against vaccination, but rather in favor of it. No vaccination is 100 percent effective. But the classics, such as the measles, mumps and rubella vaccines, are 95 percent or more effective.

Happy ending.

And how can the happy ending be topped? Not only is all of Mumpitz convinced, but the letter carrier also convinces his colleagues that, especially for people who have a lot of contact with others, the following applies: I only want to infect other people with my good mood – with nothing else.
